# “Monoallelic germline methylation and sequence variant in the promoter of the *RB1* gene: a possible constitutive epimutation in hereditary retinoblastoma”

**DOI:** 10.1186/s13148-015-0167-0

**Published:** 2016-01-08

**Authors:** Guadalupe Quiñonez-Silva, Mercedes Dávalos-Salas, Félix Recillas-Targa, Patricia Ostrosky-Wegman, Diego Arenas Aranda, Luis Benítez-Bribiesca

**Affiliations:** Hospital de Pediatría, Centro Médico Nacional Siglo XXI, IMSS, Unidad de Investigación Médica en Genética Humana, México, D.F. Mexico; Departamento de Genética Molecular, Instituto de Fisiología Celular, Universidad Nacional Autónoma de México (UNAM), México, D.F. Mexico; Laboratorio de Genómica, Instituto de Investigaciones Biomédicas, Universidad Nacional Autónoma de México (UNAM), México, D.F. Mexico; Hospital de Oncología, CMNS-XXI, IMSS, Unidad de Investigación Médica en Enfermedades Oncológicas, Av. Cuauhtémoc 330, 06725 México, D.F. Mexico

**Keywords:** *RB1*-promoter, Sequence variant, Constitutive epimutation, Methylation, Genetic-epigenetic predisposition

## Abstract

**Background:**

Retinoblastoma is a malignant tumor of the retina in children <5 years of age and occurs after two mutations in the *RB1* gene. The first mutation (M1) is germinal and confers predisposition to the hereditary type, which is transmitted as an autosomal dominant highly penetrant trait, so 90 % of carriers develop retinoblastoma; however, 10 % of carriers either do not develop the tumor or develop it unilaterally. Most mutations are point mutations. Inactivation of the RB1 gene is usually caused by mutations affecting the coding region. Silencing by methylation of the *RB1* promoter has been observed in retinoblastoma tumors as a second mutation (M2) and is classified as somatic epimutation. Germline methylation of the *RB1* gene promoter was studied in a particular pedigree of six generations from the paternal side, with incomplete penetrance and bias towards healthy male carriers and those affected with unilateral retinoblastoma.

**Results:**

The methylation status of the 27 CpGs dinucleotides that constitute the core of the RB1 gene promoter, analyzed by cloning and genomic sequencing after DNA sodium bisulfite conversion, demonstrated a monoallelic methylation pattern which coincides with a c. [−187T > G; −188T > G] sequence variant that is found in peripheral blood lymphocytes and tumor DNA. Unexpectedly, it was the mother who transmitted this variant to two more generations. Microsatellite markers of D chromosome showed a biparental contribution of both D13 chromosomes to the retinoblastoma phenotype, conferring double heterozygosity in the affected cases.

**Conclusions:**

The monoallelic genetic-epigenetic finding, the sequence variant, and methylation suggest a constitutive epimutation and probably a genetic-epigenetic hereditary predisposition for retinoblastoma in this family.

## Background

Retinoblastoma (Rb) is a malignant tumor of the eye that originates from the developing retina in children <5 years of age. Its incidence is 1:15,000 to 1:20,000 live births [1]; 40 % of the cases are hereditary (10 % are due to a germline mutation in the tumor suppressor *RB1*gene (13q14) transmitted by one of the affected parents and 30 % are due to a de novo germline mutation). The remaining 60 % of cases are sporadic [2, 3]. The germline mutation M1 is transmitted as an autosomal dominant trait with high penetrance; 90 % of mutation carriers develop bilateral Rb, whereas the remaining 10 % are frequently asymptomatic or they show unilateral Rb (incomplete penetrance (IP)) [3]. The tumor is developed with the second mutation (M2) in somatic tissue. In 70 % of the cases, M2 is due to a loss of heterozygosity (LOH) [2, 3].

In the *RB1* gene, a very wide range of mutations has been described in literature. Many of these mutations are cryptic, which makes their identification difficult. Haplotype analysis with polymorphic markers in the *RB1* gene or on chromosome 13 is useful in determining the parental origin of the mutant chromosome, especially in cases in which the mutation has not been identified [4]. Progenitor-specific effects have been identified in patients with Rb such as preferential retention of paternal alleles in tumors and distortion in the transmission of the trait among the offspring of affected males [5, 6].

Kanber et al. [7] showed that *RB1* is an “imprinted” gene, preferentially expressed by the maternal allele. The imprinted expression of *RB1* is associated with the differential methylation of a CpG island in the intron 2 of this gene.

The first report by Greger in 1989 [8] on the silencing by methylation of the *RB1* promoter in retinoblastomas noted the importance of the methylation silencing of the promoter of a tumor suppressor gene in oncogenesis. Subsequent studies have documented methylation of the 5′ region of the *RB1* gene in retinoblastoma [9]. Ohtani-Fujita et al. [10] showed that in vitro methylation of the *RB1* promoter decreased the expression of pRB. They identified two transcription factors that do not bind to *RB1* when the recognition sequence is methylated.

De la Rosa-Velázquez et al. [11] demonstrated that *RB1* is silenced by methylation of the promoter region and that the CTCF nuclear factor protects this region from methylation. Dávalos-Salas et al. [12] reported that ablation or blockage of the CTCF recognition sites in *RB1* promoter leads to quick and consistent gene silencing.

Silencing of the *RB1* promoter by methylation has been observed in both sporadic and hereditary Rb tumors [9, 13, 14]. However, this alteration has not been found in peripheral blood lymphocytes from those patients, and it has been classified as a somatic epimutation [14].

In this study, we explored the possibility of the germline methylation of the *RB1* gene promoter in a family that shows incomplete penetrance (IP) in a six generation genealogy. Furthermore, in this family, there is a gender bias because there are more males showing unilateral Rb in comparison to the number of females suffering from the same condition. These findings were unexpected.

## Results

### Inheritance pattern

**Fig. 1:**
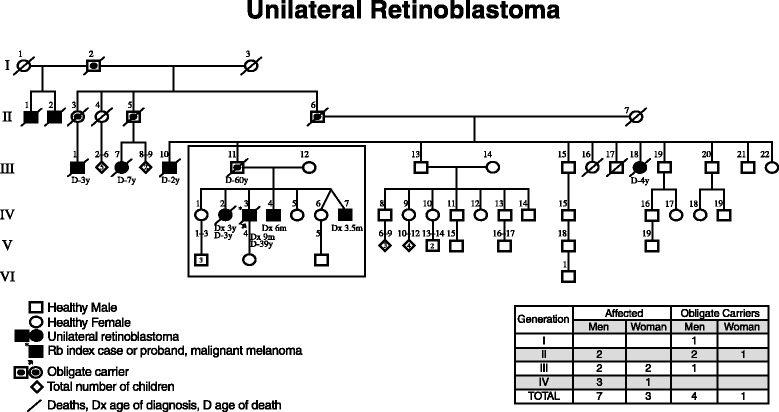
Shows the genealogy of six generations (I–VI) where generations III–V of the RB-F60 nuclear family is framed

Figure 1 shows the genealogy of six generations (I–VI) where generations III–V of the RB-F60 nuclear family are framed. The index case (↑) IV-3 was a 38-year-old patient diagnosed with malignant melanoma in the cervical region as a second neoplasm—one of the most important cause-specific mortality in long-term survivors of hereditary Rb [15]. He was identified during the aforementioned screening. The patient developed unilateral right eye Rb (OD) at 9 months of age, the same as three of his six siblings (IV-2, IV-4, and IV-7) who were affected with left eye unilateral Rb. Case IV-2 was diagnosed and died at 3 years of age; in IV-4 and IV-7, diagnoses were made at 6 and 3 1/2 months old, respectively. None of the affected patients presented associated congenital malformations.

The distribution and total number of affected patients and obligate carriers are indicated for every generation. The first obligate carrier was a healthy male from generation I (I-2), the two children from his first marriage were affected males who died (II-1 and II-2). In his second marriage he transmitted the mutation to three successive generations through four healthy carriers (II-3, II-5, II-6 and III-11). The male/female ratio was 3:1. The five carriers are the parents of ten affected descendants (seven males and three females) with unilateral Rb. The ratio of affected males/females is 2.3:1.

Thirty-three percent of I-2 children (II-1 and II-2) are affected; 50 % are healthy obligate carriers (II-3, II-5, and II-6). Carrier II-3 had only one male child who was affected and died. The percentage of II-5 affected children was 33 % (1/3 children), and in II-6, 18 % (2/11) were affected. In contrast, the percentage of affected children of III-11 (healthy carrier and obligate transmitter of RBF-60) was 57 % (4/7 children).

The total number of family members showing Rb is found in the lower box of Fig. 1 (seven males and three females) as well as the obligate carriers (four males and one female). The overall male/female ratio is 2.75:1.

### Methylation pattern

1. The methylation status of the 27 CpGs dinucleotides that constitute the core of the *RB1* gene promoter was analyzed by sodium bisulfite-treated DNA followed by cloning and genomic sequencing. Bisulfite converts all unmethylated cytosines to uracils. The only remaining cytosines are derived from methylated cytosines in the genomic sequence [13]. A heterogeneous pattern of DNA methylation in peripheral blood lymphocytes (PBL) from the mother, the index case and his siblings, and his daughter was observed through two generations. This pattern was also found in the melanoma of the index case.
2. In 16/27 CpGs of the *RB1* promoter (1, 2, 3, 5, 8, 11, 13, 14–16, 19, 22–24, 26, and 27), methylation was systematically observed. The presence of methylation was found in clones that we denominate “C” because they present two base changes in positions: c. [−187T > G; −188T > G] upstream of the initiating codon [16] (GenBank Accession L11910.1 GI: 292420) between the 3′ CpG 17 recognition sequence of the activating transcription factor (ATF) [17] and the 5′ E2F sequence [18] aaGTGACG**T T**TTCCCGCG changing to aaGTGACG**G G**TTCCCGCG. This change in sequence is shown in Fig. 2.

**Fig. 2:**
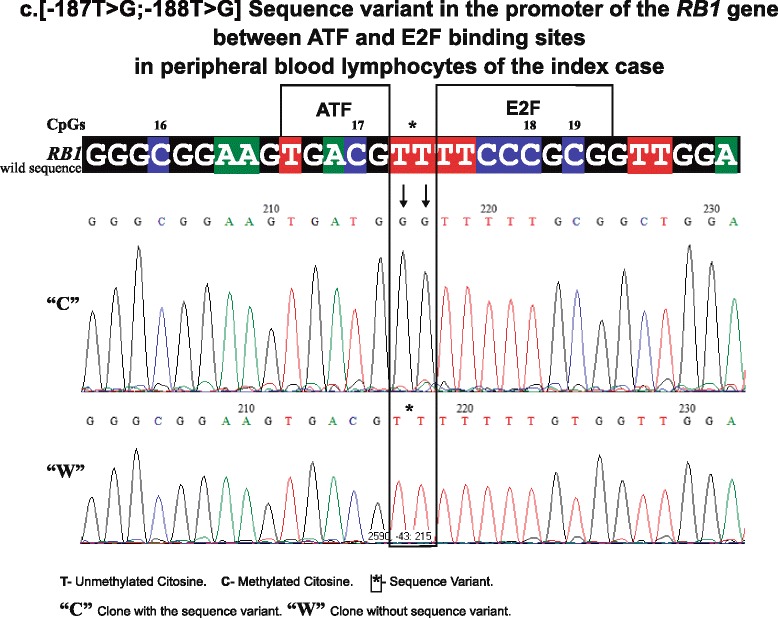
Shows a representative electropherogram of “C” clone with the sequence variant and “W” clone without the sequence variant in PBL of the index case (the sequence variant between ATF- and E2F-binding sites changes TT bases of the wild sequence to GG in “C” clones). The CpGs 16 to 19 are included
3. Clones we denominate “W” were observed with scarce methylation or methylation in CpGs different from those in “C” clones.
4. The percentage of “C” clones with the c. [−187T > G; −188T> G] change in each analyzed sample, fluctuating between 47 and 100 %. In the nine analyzed samples, basically two methylation patterns were identified, dependent on the percentage of “C” clones in each sample. These patterns are shown in Fig. 3a, b and respectively correspond to the melanoma and PBL of the index case. In each of these patterns, the 27 CpGs of the *RB1* promoter are shown, pointing out those CpGs in which transcription factors bind as well as the methylated CpGs.

**Fig. 3:**
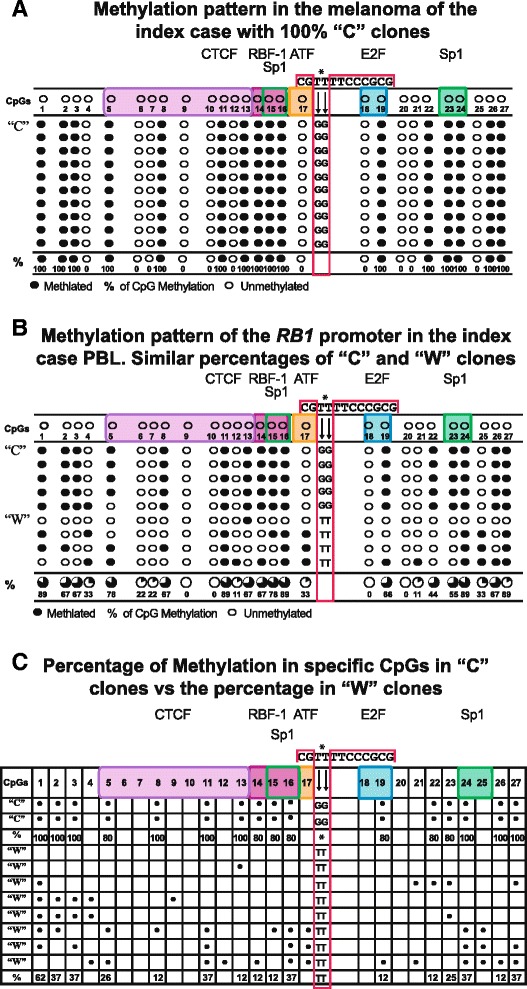
**a** This pattern of methylation found in the melanoma suggests hemizigosity which probably corresponds to the presence of the maternal-methylate allele and to the absence (loss of heterozygosity) of the paternal allele. This pattern contrasts with that in **b** observed in PBL of the index case and his siblings which suggests monoallelic methylation. **c** The bracket included clearly shows the difference of methylation between “C” and “W” clones

Figure 3a corresponds to a pattern with 100 % “C” clones showing methylation in the mentioned 16 CpGs. This pattern was observed in the DNA from the melanoma of the index case suggesting hemizigosity. Figure 3b shows a mixed pattern and equivalent percentages with 50 % “C” clones and 50 % “W” clones, suggesting monoallelic methylation. This pattern was observed in all family members including the index case and his daughter.

Figure 3c shows schematically the contrast between the methylation status among the 27 CpGs of the “C” clones and the “W” clones. The “W” clones in this figure were selected from the total analyzed samples to clearly show the differences in methylation and highlighting the contrast of methylated CpGs between the two clones. It is observed that the methylation pattern of the “C” clones systematically involves specific CpGs where *RB1* key transcription factors bind. One hundred percent of these clones showed methylation in four of the nine CpGs: 5, 8, 11, and 13, where CTCF binds [11, 12] in the 14, 15, and 16 CpGs corresponding to the consensus sequence where the RBF1 and Sp1 binding sites overlap [17, 19]; and methylation in CpG 19 is one of the two CpGs where E2F binds [18] in addition to the methylation in the Sp1 CpGs 23-24 recognition sites [20].

In contrast, in the “W” clones, methylation is observed in CpGs where no transcription factors bind or the percentage that coincides with CpGs of the “C” clones is remarkably low, as shown in the schematic representation of Fig. 3c.

### Segregation of polymorphic microsatellites

**Fig. 4:**
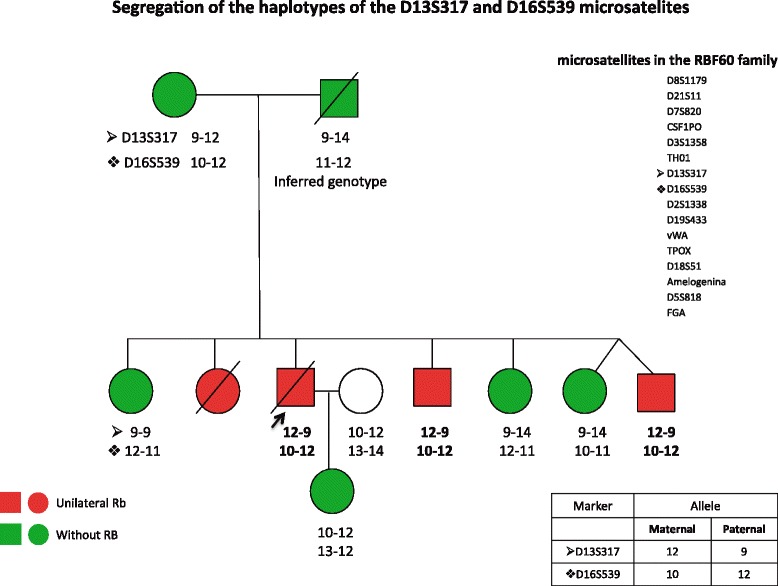
Displays the specific segregation of haplotypes of the D13S317 and D16S539 microsatellites in members of RBF60 family that show unilateral Rb

Due to the father’s death (transmitter of the Rb trait), the corresponding analysis was unable to be performed. The paternal genotype was deduced from the genotype found in his offspring. Segregation of all the analyzed markers showed biological certainty of kinship between parents and children (haplotypes not shown). Figure 4 shows the genealogy of the nuclear family, and the analyzed microsatellite markers are listed. The haplotype corresponding to markers D13S317 (chromosome 13 q31.1) [21] and D16S539 (chromosome 16 q14.1) [22] in each of the family members is noted. The difference of haplotypes in these markers among those affected with Rb vs. the haplotype of these markers in the other family members is highlighted as shown in the box on the right of Fig. 4.

## Discussion

The studied family shows an autosomal dominant pattern characteristic of hereditary Rb with several family members affected by the condition in each generation [3]. However, the number of affected members (18 and 33 %) in generations II and III is <50 % as expected in this type of inheritance. The higher number of affected males with respect to females also deviates from this ratio. On the other hand, none of the 10 obligate carriers developed Rb and all of those affected have unilateral Rb. These familial data indicate that this corresponds to the small but significant number of families in which Rb is inherited with incomplete penetrance (IP) [3].

In contrast to what was observed in generations II and III, in generation IV, corresponding to the family of this study, 4/7 children (57 %) were affected, showing a change to complete penetrance. However, the moderate expression of unilateral tumors is preserved as well as the bias of affected males.

It has been suggested that these biases are due to a higher mutation rate in spermatogenesis than in oogenesis, meiotic drift, and to the existence of imprinted genes [4–7, 23]. Klutz et al. [24] studied two non-related families with Rb and IP who carried the same mutation. This showed variation in the phenotypic expression of Rb and a higher number of affected members when the father was identified as the transmitter of the mutant allele.

To identify the paternal germline mutation that has conditioned IP in the reported family, complete sequencing of *RB1* would be required because the type of mutations in the families with IP are not part of the spectrum of germline mutations found in most families with Rb and almost every family has its own mutation [25, 26]. The mutations conferring IP in general cause a quantitative decrease in the expression or a partial loss of the *RB1* suppressor function [3, 10, 17, 24]. It has been suggested that in families with IP mutations, LOH is oncogenically insufficient because the homozygosity of the predisposing allele still retains suppressive activity and the carriers would be asymptomatic [17, 20]. For Rb development, a mutation with complete loss of function in the normal allele is required [17]. Because these mutations are less common (30 vs. 70 % LOH), the proportion of those affected in these families may be lower [3, 17]. This would explain the lower number of those affected in generations II and III. Another explanation for the lower number of those affected may be related to the differential expression of *RB1*, consecutive to its normal imprinting state [7]. In this case, the preferential expression of the maternal allele could substitute the low expression of the putative paternal germline IP mutation, which would avoid the development of Rb in asymptomatic carriers of this family.

Regarding the change of penetrance specifically in the RB-F60 nuclear family, the possibility that could help explain this change is the finding of the c. −[187T > G; 188T > G] sequence variant between the ATF and E2F sequences. In the first, Sakai et al.[17] found the G > T transversion (position 189 upstream from the start codon) in a family with hereditary Rb and IP, which allowed identifying the binding site of this nuclear factor in the core of the *RB1* promoter, necessary for transcriptional activation of *RB1* and the oncogenic suppression [17]. Whereas E2F is involved in gene repression [18], studies with transgenic reporters have shown that mutations at a single E2F site are critical for gene repression, further suggesting that this factor may contribute to the regulation of the transcription of *RB1* [27]. The change observed in this study is found in positions 187 and 188, and the transversion is also different. Mutations in the *RB1* promoter are rare. In the ATF sequence, three cases of mutations were found [4, 25, 26], whereas in E2F, no similar reports were found [25, 26].

Three aspects stand out in reference to the sequence variant observed in this family: (a) it involves two adjacent bases, one in an activator site and the other in a repressor site; this variant was not found reported [25, 26]; (b) the variant was unexpectedly found in PBL DNA from the index case’s mother, who transmitted it to two generations including all their children (generation IV) and the index case’s daughter (generation V), suggesting germline occurrence that has been segregated with a dominant inheritance pattern; (c) and it coincides or is associated with methylation mainly of recognition sequences for transcription factors in the *RB1* promoter. Methylation is apparently allele-specific because, in general, clones without this change do not show consistent methylation.

Both the ATF sequence mutations and the methylation of the promoter are oncogenic [9, 10, 13, 17], suggesting that this sequence variant associated with methylation could also be oncogenic.

On the other hand, the methylation pattern seen in PBL in this family has similarities with the methylation pattern reported by Stirzaker et al. [13] in Rb tumors. This shows methylation in the 27 CpGs of the *RB1* promoter, including binding CpGs to transcription factors but with varying methylation density both among the CpGs and from tumor to tumor. Some individual CpGs were unmethylated, highlighting the CpG from E2F. In our study, extensive methylation is also observed but is more consistent in the CpGs where transcription factors bind, although the CpG in ATF was found unmethylated as well as one of the two CpGs in E2F. However, it is not possible to perform a quantitative comparison of methylation in specific CpGs because no quantification was performed as in the study by Stirzaker et al.

It is interesting to compare the results with the findings of Dávalos et al. [12] in Fig. 4c regarding the methylation pattern of cultured cells in which the CTCF binding sites were mutated in the *RB1* gene promoter. It was demonstrated that CTCF protects the promoter from methylation. There are striking similarities in the methylation pattern in the promoter from the studied family with the methylation pattern of the cells lacking CTCF protection, which also showed low expression levels of pRB. Similar analyses have not been performed in this family.

The silencing of *RB1* consecutive to the promoter methylation reported in Rb tumors [10, 13, 14] and the gain of methylation in the promoter of this gene consecutive to mutations in key-binding sites to transcription factors [10–12, 17, 18, 20] would allow us to suggest that in RBF60, this double finding apparently in the same allele could correspond to an epimutation consecutive to the TT > GG transversion positioned between an activating sequence and a repressor sequence as previously mentioned.

This supposition is sustainable on the basis that in some neoplastic diseases with hereditary predisposition, similar alterations to those observed in this study have been reported in which sequence variants coexist in adjacent or distant genes that promote epigenetic modifications, specifically the methylation in the promoters of specific genes [28–30]. These changes show that in the etiology of these conditions, very complex genetic-epigenetic interactions coexist and are involved in the transcriptional silencing which, among others, is consecutive to antisense transcription [31]. These mechanisms are helping to understand this new field of epigenetic inheritance and its hereditary transmission through epimutations [32, 33].

It should be emphasized that these particular types of epimutations are hereditary because the epigenetic methylation modifications are secondary to changes in cis in the gene sequences that occur at the germline level and are dominantly transmitted to offspring [28, 30, 31].

Speculatively, we suggest that the sequence variant in RBF60 according to some currently unknown mechanism induces methylation of the *RB1* promoter. This originates a constitutive epimutation [32, 33] because methylation is found in the melanoma of the index case and in the PBL. However, this finding in the mother was unexpected. In fact, she represented the control arm of the study because no obvious pathological data were found in the maternal family. As shown in the genealogy, it is clear that the transmission was only of paternal origin.

Because the father died, polymorphic markers were analyzed in an attempt to demonstrate the paternal origin of the mutant allele. However, the results suggest that both parents were carriers of germline mutations. In the father, this is still unidentified as previously mentioned but was demonstrated by the IP inheritance pattern and the mother as a carrier and transmitter of a probable constitutive epimutation in the *RB1* gene promoter.

The biparental contribution is further supported by the results obtained with the microsatellite markers because specific segregation was observed not only of one allele of the paternal D13 chromosome but also by the specificity of one of the alleles of D13 chromosome of maternal origin, which is segregated with a unique haplotype in those affected. This suggests a biparental germinal contribution of both D13 chromosomes to the Rb phenotype. A biparental-specific share of the alleles of chromosome 16 was also observed, which represents information of additional interest in this family since deletion of the long arm of this chromosome (16q) is related to a particular type of Rb [34].

## Conclusions

Affected children of RBF60 family have double heterozygous germline (M1) mutations in the *RB1* gene. The first M1 was transmitted by the father and is associated to IP and is more widespread in males. The second M1 was the germline “epimutation” (sequence variant and methylation in the *RB1* promoter) that was transmitted by the mother, which granted a higher penetrance and hereditary genetic-epigenetic predisposition for developing Rb, unlike the families of generations II and III that are only heterozygous for the first M1.

## Methods

The RBF60 family, the focus of this study, was identified through a clinical screening performed at a specialized oncology hospital to locally determine the frequency and type of familial aggregation of cancer. In patients with a history of cancer, a complete genealogical study was conducted. Familial aggregation of cancer was assessed using validated clinical criteria [35]. Diagnosis of the index case from each family was obtained by histopathology as well as diagnosis of the affected relatives or through the use of clinical records, death certificate, or family history. The proband or index case (↑) was identified; when at 38, he developed a malignant melanoma as a second neoplasm. His family was selected for molecular study to research the possibility of germline *RB1* promoter methylation for presenting a six-generation genealogy and unilateral Rb in three successive generations through healthy carriers and with a higher number of affected and health carrier males. Informed consent was granted by all family members for obtaining biological samples.

### Methylation analysis

#### Samples

DNA was extracted from PBL from all family members except from the father and an affected sister (both deceased) of the index case. For extraction and purification of DNA, a Qiagen kit was used. DNA was also extracted from the paraffin-embedded melanoma of the index case. For DNA extraction and purification, a Qiagen kit was used for tissue included in paraffin blocks following manufacturer’s instructions. DNA was eluted in water.

#### Sodium bisulfite treatment

Eight DNA samples from peripheral blood lymphocytes and one from the melanoma were processed. The reaction to sodium bisulfite conversion was performed for 16 h at 50 °C with 2 μg DNA as previously described [11–13]. Samples were purified using the Wizard DNA Clean-up Column System (Promega). DNA was precipitated with ethanol, dried, resuspended in 30 μl of water, and then stored at −20 °C.

#### PCR amplification and primers—cloning

The *RB1* promoter region constituted by an island consisting of 27 CpGs dinucleotides (position 1634-2020 GenBank accession number and version L11910.1 GI: 292420) was analyzed. PCR amplifications were performed in a 30 μl reaction mixture containing 2 μl sodium bisulfite-treated genomic DNA, 10 mM dNTPs, 15 pm primers, 2.5 mM MgCl, and 2.5 U AmpliTaq DNA polymerase. Thermal cycling conditions were as follows: 95 °C for 15 min, 95 °C for 40 s, 66 °C for 40 s, 72 °C for 40 s, and 72 °C for 5 min for 33 cycles. Previously used MIP primers (methylation independent primers) [12] were Rb forward: 5′ TTAGGTTTTTTAGTTTAATTTTTTAT. Rb reverse: 5′-AACTATAAAAAAACCCCAAAAAAAAC (the restriction site for cloning not annotated). The 300-bp amplification product was purified using the QIAEX II Gel Extraction Kit (Qiagen) and cloned using the pGEM-T Easy Vector System (Promega) following the manufacturer’s instructions. To determine the methylation pattern, eight to ten individual clones from each of the nine mentioned samples were sequenced.

### Polymorphic microsatellite analysis

Segregation of 16 microsatellite markers was analyzed: D8S1179, D21S11, D7S820, CSFIPO, D3S1358, TH01, D2S1338, D19S433, vWA, TPOX, D18S51, Amelogenin, D5818, and D16S539 FGA (chromosome 1614q21), including one from D chromosome: D13S317 (chromosome 13q31.1) [16].

## Abbreviations

IP: incomplete penetrance
LOH: loss of heterozygosity
PBL: peripheral blood lymphocytes
pRB: RB protein
Rb: retinoblastoma
