# Erratum to: “Monoallelic germline methylation and sequence variant in the promoter of the RB1 gene: a possible constitutive epimutation in hereditary retinoblastoma”

**DOI:** 10.1186/s13148-017-0323-9

**Published:** 2017-03-16

**Authors:** Guadalupe Quiñonez-Silva, Mercedes Dávalos-Salas, Félix Recillas-Targa, Patricia Ostrosky-Wegman, Diego Arenas Aranda, Luis Benítez-Bribiesca

**Affiliations:** 1 Unidad de Investigación Médica en Genética Humana, Hospital de Pediatría, Centro Médico Nacional Siglo XXI, IMSS, México, D.F. Mexico; 2 Departamento de Genética Molecular, Instituto de Fisiología Celular, Universidad Nacional Autónoma de México (UNAM), México, D.F. Mexico; 3 Laboratorio de Genómica, Instituto de Investigaciones Biomédicas, Universidad Nacional Autónoma de México (UNAM), México, D.F. Mexico; 4 Unidad de Investigación Médica en Enfermedades Oncológicas, Hospital de Oncología, CMNS-XXI, IMSS, Av. Cuauhtémoc 330, 06725 México, D.F. Mexico

Following publication of this article [1], it has come to our attention that the following publication should have acknowledged Programa de Doctorado en Ciencias Biomédicas de la Universidad Nacional Autónoma de México for supporting the work.
